# Supplementary material for: Quality Verification with a Cluster−Controlled Manufacturing System to Generate Monocyte−Derived Dendritic Cells
Source: Vaccines (Basel). 2021 May 20;9(5):533. doi: 10.3390/vaccines9050533 (PMC8160655; doi:10.3390/vaccines9050533)
Supplement: Supplementary file 1 [file vaccines-09-00533-s001.zip › vaccines-1191790-supplementary.pdf]

vaccines

Supplemental Materials

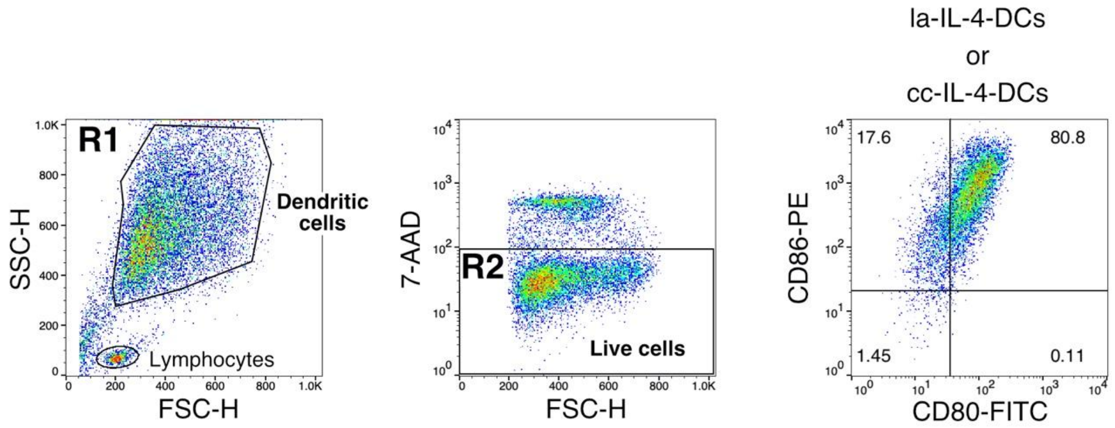

Figure S1. Gate strategy for the identification of low−adherent IL-4−DCs or cluster−controlled IL-4−DCs. The frequency of positive cells was analyzed using the Flowjo software in two regions. After staining cells with each antibody of DC maturation markers, DCs gated on forward scatter (FSC) and side scatter (SSC) without the lymphocyte population (R1). Live cells gated on FSC and 7−AAD without dead cells were examined for immunophenotyping (R2). The right panel indicates percentage of CD80/CD86 dot plot analysis.

Table S1. Cross tabulation findings on ELISpot assays and the DC phenotype in 10 cancer patients.

–		ELISpot assay		
		Negative	Positive	Total
**CD80 (%) 75th percentile**	Positive	0	2	2
	Negative	7	1	8
	Total	7	3	10
chi−square test: $\chi^2 = 5.83$, $*p = 0.016$				

–		ELISpot assay		
		Negative	Positive	Total
**CD86 (%) 75th percentile**	Positive	1	2	3
	Negative	6	1	7
	Total	7	3	10
chi−square test: $\chi^2 = 2.74$, $p = 0.097$				

–		ELISpot assay		
		Negative	Positive	Total
**CD83 (%) 75th percentile**	Positive	0	2	2
	Negative	7	1	8
	Total	7	3	10
chi−square test: $\chi^2 = 5.83$, $*p = 0.016$				

–		ELISpot assay		
		Negative	Positive	Total
**CD40 (%) 75th percentile**	Positive	1	1	2
	Negative	6	2	8
	Total	7	3	10
chi−square test: $\chi^2 = 0.48$, $p = 0.49$				

The number of patients in each category is shown. ELISpot assay data were divided in 75th percentile DC phenotypic panels. At least 15 WT1−specific spots per 1×10^6 PBMCs in the ELISpot assays were defined as positive as described as previously [40]. DC maturation markers were grouped more than or less than 75th percentile. Statistical significance test was done by chi−square test. *, *p*−value < 0.05.

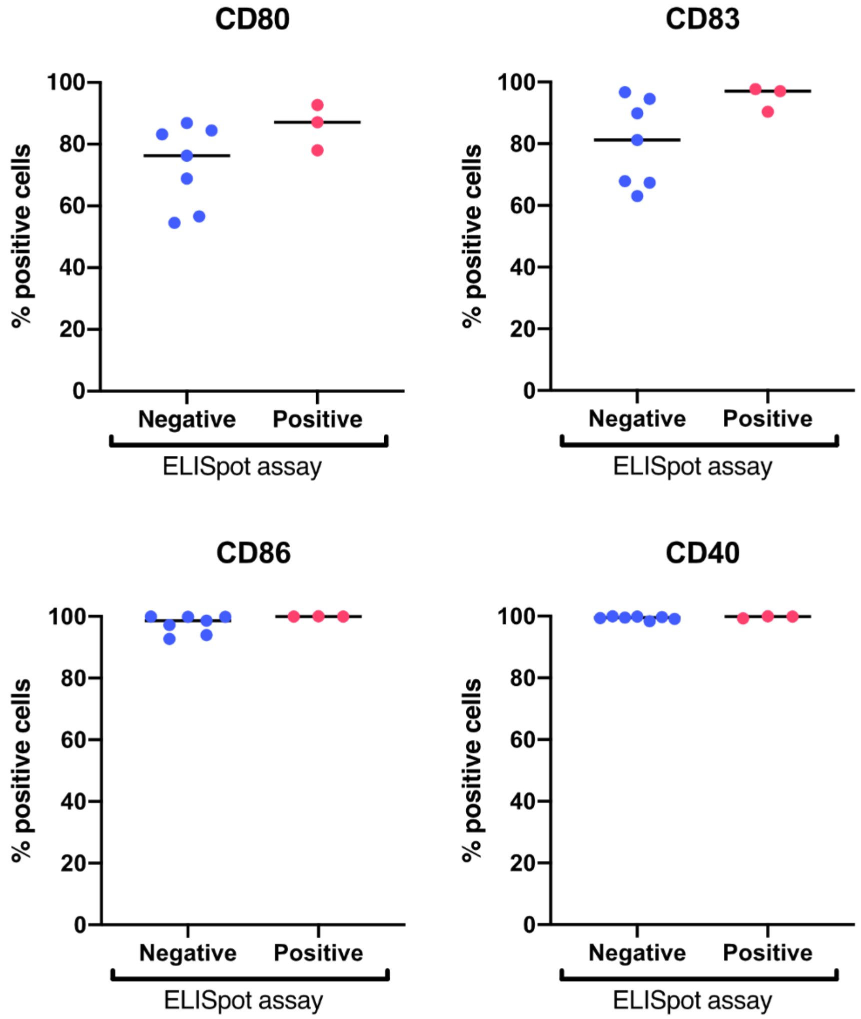

Figure S2. The comparison of DC maturation markers in the groups detected with ELISpot assays. Patients with HLA−A*24:02 underwent a WT1 peptide−pulsed DC therapy (n=10). At least 15 WT1−specific spots per 1 × 10^6 PBMCs were defined as positive using ELISpot assays after one course of DC vaccination. The DC phenotypes from the same patients indicate CD80, CD86, CD83 and CD40. The population of positive cells was determined in propidium iodide (PI)−negative and DC−gated populations excluding lymphocytes from forward and side scatter.

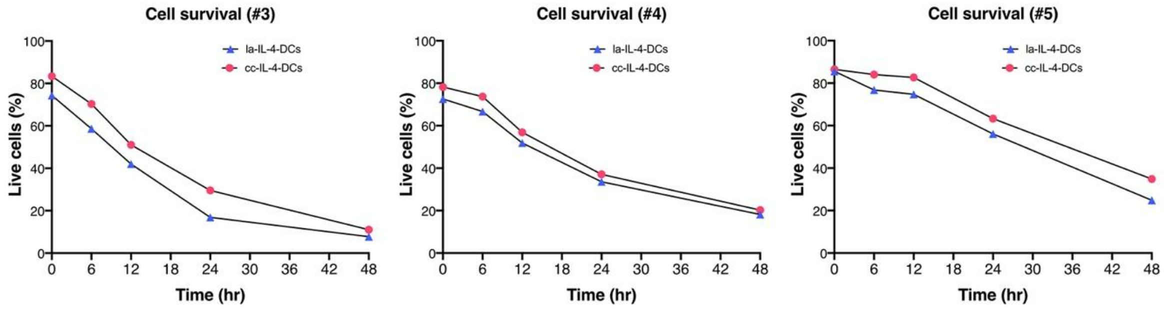

Figure S3. The analysis of cell survival in low−adherent IL-4−DCs and cluster−controlled IL-4−DCs. Frozen mature DCs were thawed and washed with saline, and then suspended in saline at 1 x 10^7 cells/ml. Cell survival rate was measured at each timepoint by trypan blue staining (n = 3).

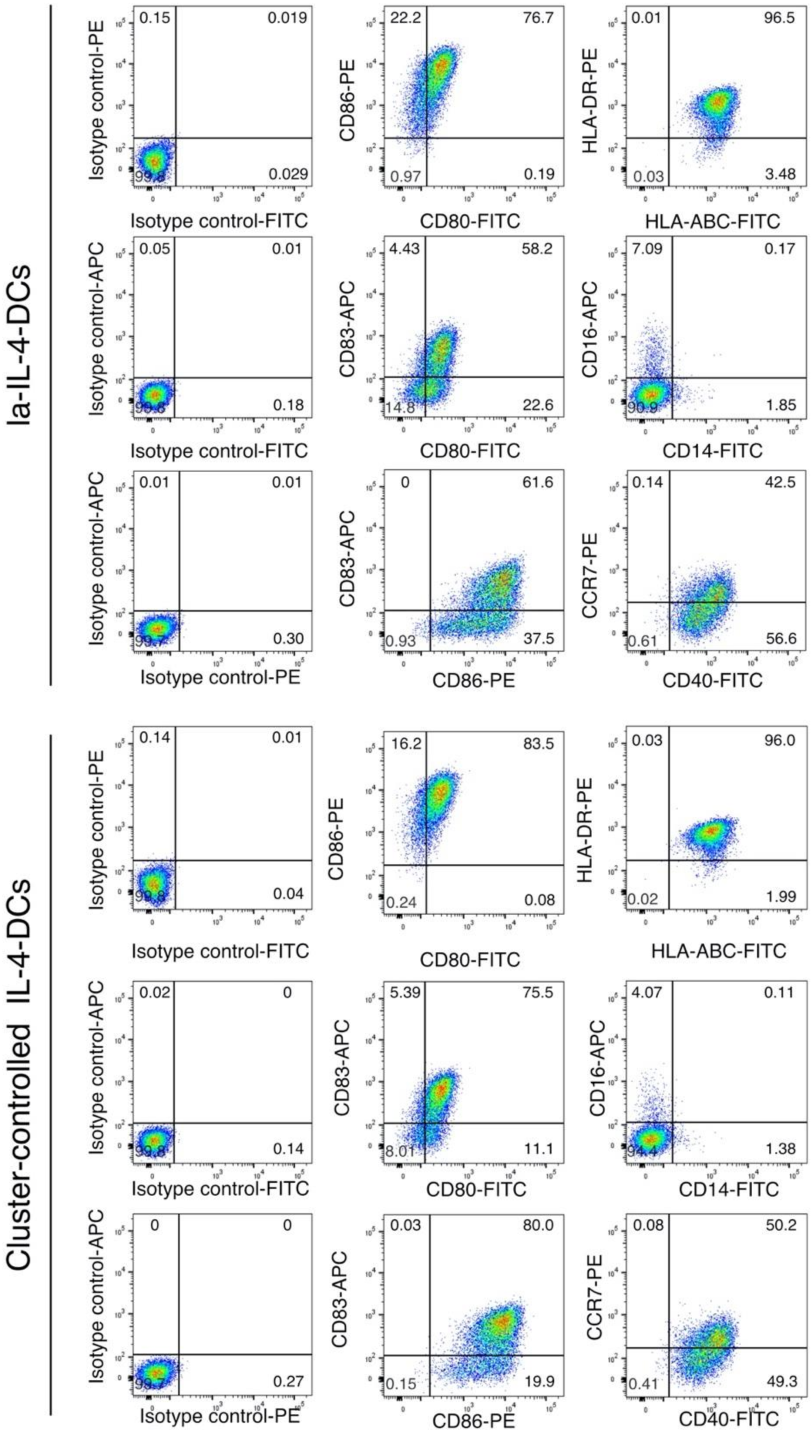

Figure S4. Phenotypic change of surface markers using a cluster−control dish for DC maturation. Dot plots of representative IL-4−DC−related markers are shown. Quadrant gate was determined so that the percentage of each isotype control as more than 99%. The numbers in the four corners of the panel indicate the positive rate for each fraction.
